# Lyc. in Bronchitis

**Published:** 1882-11

**Authors:** F. Bruns

**Affiliations:** Boston


### LYC. IN BRONCHITIS.

Dr. F. Bruns, M. D., Boston.

My horse had to be led out of her burning stable on the 8th of August, and took, probably, some cold. On the evening of the same day I was informed that the animal was not well. I found her somewhat feverish. I tried to give her some Acon., but did not succeed. The next morning I was called out of bed and found her lying on her right side, and had fearful dyspnœa. The breathing was from 50 to 60 in the minute. I knew Acon. would not save the life of the horse now, but gave it to ameliorate the fever. It lessened the number of breathing to 40 in a minute, but did not in the least lessen the obstruction in the capillaries and larger bronchi. I led her out of her place to make a closer examination. The animal tottered to the watering trough, put her nose over the water to inhale the cold evaporation. Every muscle moved so forcibly that her flanks not only, but her hardened epithelium shuddered, nostrils moved like wings. Lyc. D m m (Swan) was given. The horse was led back to her place. Improvement was noticed in a quarter of an hour, and in three hours the horse was free. She asked the next day for more to eat by pawing. Yesterday, six days after being taken sick, I drove her the first time, and noticed her only coughing very slightly a few times. Repetition of dose? I never repeat as long as improvement continues. I have treated chronic diseases only for the past three years and cured most of my patients by only two or three remedies, which were administered as Hahnemann, Bœnninghausen, Jahr, etc., have told us. I am no friend of wild-goose chases.

